# Supplementary material for: Mechanisms governing the pioneering and redistribution capabilities of the non-classical pioneer PU.1
Source: Nat Commun. 2020 Jan 21;11:402. doi: 10.1038/s41467-019-13960-2 (PMC6972792; doi:10.1038/s41467-019-13960-2)
Supplement: Supplementary file 7 — Source data [file 41467_2019_13960_MOESM7_ESM.zip › Source_Data/FigureS3/FigureS3H_MotifScanOutput/homerResults/motif32.similar.html]

motif32

## Information for motif32

A
C
T
G
C
G
T
A
C
G
A
T
A
C
G
T
C
T
A
G
A
G
C
T
A
C
G
T
C
G
T
A
A
C
G
T
A
G
T
C
A
G
C
T
  
Reverse Opposite:  

C
T
G
A
A
C
T
G
C
G
T
A
A
C
G
T
G
T
C
A
C
T
G
A
A
G
T
C
C
G
T
A
C
G
T
A
A
C
G
T
A
G
T
C
  

|  |  |
| --- | --- |
| p-value: | 1e-25 |
| log p-value: | -5.842e+01 |
| Information Content per bp: | 1.936 |
| Number of Target Sequences with motif | 35.0 |
| Percentage of Target Sequences with motif | 0.91% |
| Number of Background Sequences with motif | 18.3 |
| Percentage of Background Sequences with motif | 0.04% |
| Average Position of motif in Targets | 204.5 +/- 173.7bp |
| Average Position of motif in Background | 144.2 +/- 107.7bp |
| Strand Bias (log2 ratio + to - strand density) | 0.1 |
| Multiplicity (# of sites on avg that occur together) | 1.00 |
| Motif File: | file (matrix) reverse opposite |

### Similar de novo motifs found

|  |  |  |  |  |  |  |  |
| --- | --- | --- | --- | --- | --- | --- | --- |
| Rank | Match Score | Redundant Motif | P-value | log P-value | % of Targets | % of Background | Motif file |
| 1 | 0.950 | C T A G C G T A C G A T A C G T C T A G C G A T A C G T C G T A A C G T A G T C A C G T A G T C | 1e-23 | -54.177350 | 0.85% | 0.04% | motif file (matrix) |
| 2 | 0.882 | C T A G C G T A A C T G C G T A A C T G C G T A A C G T G T C A C G T A A G T C C G T A C G T A A C G T A G T C | 1e-21 | -48.854516 | 0.59% | 0.01% | motif file (matrix) |
